# A composite score based on immune-related gene prognostic index and m^6^A risk score of head and neck squamous cell carcinoma

**DOI:** 10.3389/fgene.2023.1061569

**Published:** 2023-02-09

**Authors:** Yizhou Yang, Zeman Cai, Kaichun Huang, Mei Li, Xiao Wang, Yinbing Lin, Sijie Chen, Zhining Yang, Zhixiong Lin

**Affiliations:** ^1^ Department of Radiation Oncology, Cancer Hospital of Shantou University Medical College, Shantou, Guangdong, China; ^2^ Nasopharyngeal Carcinoma Research Center of Shantou University Medical College, Shantou, Guangdong, China; ^3^ Shantou University Medical College, Shantou, Guangdong, China

**Keywords:** head and neck squamous cell carcinoma, m^6^A, immunotherapy, tumor immune microenvironment, prognosis

## Abstract

**Background:** Immunotherapy has been demonstrated favorable in head and neck squamous cell carcinoma (HNSCC). Studies indicated that immune-related gene prognostic index (IRGPI) was a robust signature, and N^6^-methyladenosine (m^6^A) methylation had a significant impact on the tumor immune microenvironment (TIME) and immunotherapy of head and neck squamous cell carcinoma. Thus, combining indicated that immune-related gene prognostic index with m^6^A status should offer a better predictive power for immune responses.

**Methods:** Head and neck squamous cell carcinoma samples from the cancer genome atlas (TCGA, *n* = 498) and gene expression omnibus database (GSE65858, *n* = 270) were used in this study. Cox regression analysis was used to construct the indicated that immune-related gene prognostic index through immune-related hub genes which were identified by weighted gene co-expression network analysis (WGCNA). The m^6^A risk score was constructed by least absolute shrinkage and selection operator (LASSO) regression analysis. Principal component analysis was used to construct a composite score, and systematically correlate subgroups according to tumor immune microenvironment cell-infiltrating characteristics.

**Results:** A composite score was determined based on indicated that immune-related gene prognostic index and m^6^A risk score. Head and neck squamous cell carcinoma patients in the cancer genome atlas were divided into four subgroups: A (IRGPI-High&m^6^A-risk-High, *n* = 127), B (IRGPI-High&m^6^A-risk-Low, *n* = 99), C (IRGPI-Low&m^6^A-risk-High, *n* = 99), and D (IRGPI-Low&m^6^A-risk-Low, *n* = 128), and overall survival (OS) was significantly different between subgroups (*p* < 0.001). The characteristics of tumor immune microenvironment cell infiltration in the four subgroups were significantly different in subgroups (*p* < 0.05). The receiver operating characteristic (ROC) curves show the predictive value of composite score for overall survival was superior to other scores.

**Conclusion:** The composite score is a promising prognostic signature which might distinguish immune and molecular characteristics, predict prognosis, and guide more effective immunotherapeutic strategies for head and neck squamous cell carcinoma.

## Introduction

Treatment of head and neck squamous cell carcinoma (HNSCC) remains challenging (Mody et al., 2021). Immunotherapy, including immune checkpoint inhibitor therapy (anti-PD-1/L1, anti-CTLA-4, etc.), chimeric antigen receptor macrophages (CAR-M), and anti-tumor-associated macrophages (anti-TAMs), has shown promise in treatment of HNSCC, but only a small percentage of HNSCC patients have sustained immune responses (Evrard et al., 2019; Malfitano et al., 2020; Cheng et al., 2021; Sloas et al., 2021; Fasano et al., 2022; Poulose and Kainickal, 2022). Many studies have shown that immune-related gene prognostic index (IRGPI) may be a potential signature of immunotherapy for a variety of tumors. However, the existing IRGPI is not detailed enough for tumor immune microenvironment (TIME) stratification, and the accuracy of prognosis prediction needs to be further studied (Chen et al., 2021; Yao et al., 2022). Methylation to form N6-methyladenosine (m6A), as the most common modification in eukaryotic RNA, plays an either positive or negative important role on RNA synthesis, transport, and translation, indirectly affects IRGPI, TIME and immunotherapy of HNSCC (Yi et al., 2020; Jing et al., 2021). However, incomplete evaluation of m^6^A regulators and inadequate detailed risk stratification remain issues to be solved (Yi et al., 2020; Chen et al., 2021; Elmusrati et al., 2021; Mody et al., 2021). Consequently, we need to identify more accurately prognostic signatures associated with therapeutic benefits to guide individualized immunotherapy for HNSCC patients.

In this study, we develop a composite HNSCC prognostic signature based on IRGPI and m^6^A risk score, and explore the molecular and immunological characteristics of diverse subgroups of IRGPI, m^6^A risk score and composite score. They then were compared with microsatellite instability (MSI), tumor immune dysfunction and exclusion (TIDE), tumor inflammation signature (TIS), and other signatures.

## Materials and methods

### Data acquisition

Samples (*n* = 498, 455 tumor samples and 43 para-cancer samples) of HNSCC patients (*n* = 454) with RNA-seq data (FPKM value), miRNA-seq data, copy number variation (CNV) and well-defined clinical stages were screened from The Cancer Genome Atlas (TCGA) (https://portal.gdc.cancer.gov). We downloaded simple nucleotide mutations in tumor samples from 451 patients. RNA-seq data and survival information of 270 HNSCC samples (GSE65858) were downloaded from the Gene Expression Omnibus (GEO) database (https://www.ncbi.nlm.nih.gov/geo). Lists of immune-related genes were downloaded from the InnateDB (https://www.innatedb.ca/) and ImmPort (https://www.immport.org/shared/home) databases.

### Identification of differentially-expressed genes (DEGs)

The RNA-seq of 498 samples (454 tumors vs. 43 para-cancer samples) obtained from TCGA was analyzed for genetic differences by the Wilcoxon test, and miRNA-seq data was analyzed using the edgeR package of R. Those genes with |log2FC| > 1 and false discovery rate (FDR) < 0.05 were considered as DEGs. After extracting the DEGs of immune-related genes obtained from ImmPort and InnateDB databases, Gene Ontology (GO) and Kyoto Encyclopedia of Genes and Genomes (KEGG) enrichment analyses were performed on immune-related DEGs by using the clusterProfiler package of R.

### Identification of immune-related hub genes

WGCNA package of R was used to perform weighted gene co-expression network analysis (WGCNA) for immune-related DEGs. In order to identify hub genes, 498 HNSCC samples were clustered to detect outliers based on the immune-related DEG expression matrix, and the similarity matrix was constructed after excluding outlier data (cut-off value 36,000). Then, the similarity matrix was converted into the adjacency matrix with soft threshold *β* = 7 and signed network type, and topological overlap measure (TOM) was used to calculate the degree of association between genes and the adjacency matrix was converted into TOM similarity matrix. After that, the TOM similarity matrix was transformed into a dissimilarity matrix based on 1-TOM, and a dynamic pruning tree was constructed to identify the modules. Finally, we set the minimum cluster size to 25 and the merge threshold function to 0.25 to separate the six modules. The igraph package of R was used to construct a gene co-expression network for genes with edge weights greater than 0.2 in the module of interest (turquoise module). After batch adjustment for biotechnological biases, using the ComBat algorithm of the sva package in TCGA and GEO cohorts, univariate Cox regression analysis was used to identify immune-related hub genes that were associated with overall survival (OS).

### Construction and validation of the IRGPI

Genes with significant impact on OS were selected from immune-related hub genes by multivariate Cox regression analysis. The IRGPI of each sample was calculated by multiplying the expression values of specific genes by their weighting in the Cox model, and then summed. Survival analysis was used to evaluate the predicting prognostic ability of IRGPI in TCGA and GEO cohorts. Patients were divided into IRGPI-high and IRGPI-low groups based on the median.

### m^6^A RNA methylation regulators

According to published studies, we collected 29 regulators of m^6^A RNA methylation, including 16 readers “YTHDC1, YTHDC2, IGF2BP1, IGF2BP2, IGF2BP3, YTHDF1, YTHDF2, YTHDF3, HNRNPA2B1, HNRNPC, RBMX, FMR1, LRPPRC, ELAVL1, EIF3A, and PRRC2A”, 10 writers “METTL3, METTL5, METTL14, METTL16, WTAP, VIRMA, RBM15, RBM15B, ZC3H13, and CBLL1” and three erasers “FTO, ALKBH3, and ALKBH5”.

### Calculation of m^6^A risk score

Based on the gene expression of 29 regulators of m^6^A RNA methylation, the survival package of R was used for univariate Cox regression analysis to construct a prognostic network map. The glmnet package of R was used for the least absolute shrinkage and selection operator (LASSO) regression analysis. After simulation 1,000 times and cross-validation, the m^6^A regulators that strongly correlated with prognosis and their coefficients were obtained. Then, the m^6^A risk score was calculated by multiplying each obtained coefficient by the corresponding m^6^A regulator expression and summing the total values. HNSCC patients were divided into m^6^A-risk-high and m^6^A-risk-low groups according to the median value, and the differences in OS between the two groups were assessed.

### Classification and composite score

Four subgroups were classified by pairwise combination of IRGPI and m^6^A risk score. IRGPI and m^6^A risk score were standardized by using the descriptives function of SPSS, and their weights were calculated by the principal component method in factor analysis. The composite score equaled the sum of the normalized IRGPI and m^6^A risk score multiplied by their corresponding weights. Univariate and multivariate Cox regression analyses were performed to verify the independent prognostic value of IRGPI, m^6^A risk score and composite score, and their relevant gene signatures were constructed by principal component analysis (PCA), with principal components 1, 2, and 3 selected as signature scores. The chi-square test (Monte-Carlo algorithm) was applied to test the correlation between clinicopathological features and composite score subgroups. Based on the interaction between mRNA and miRNA in the ENCORI database (http://starbase.sysu.edu.cn/) (Li et al., 2014), we used Cytoscape (v3.8.2) to construct the target interaction network of differentially-expressed miRNAs and mRNAs that were expressed by the genes used to construct the three scores. To further explore the interrelationships between proteins expressed by the genes reflected in the scores, we used the STRING database (https://www.string-db.org/) to construct the protein-protein interaction (PPI) network.

### Comprehensive analysis of the molecular immunological characteristics and immunotherapy in subgroups

Based on the DEGs, gene set enrichment analysis (GSEA) was applied to the composite score subgroups with the GO, KEGG and HALLMARK gene sets, which were downloaded from the GSEA database (http://www.gsea-msigdb.org/gsea/index.jsp) using the clusterProfiler package of R. The number and quality of genetic mutations in subgroup tumor samples was analyzed by using the maftools package of R. Correlation analysis of the composite score with PD-1/L1, CTLA-4 and tumor mutation burden (TMB) were performed. To identify the immune characteristics of 454 HNSCC samples, we analyzed their RNA-seq data by using the CIBERSORT tool (https://cibersort.stanford.edu/) and performed 1,000 iterations to estimate the relative proportions of 22 kinds of immune cells among subgroups. We analyzed the expression differences of 22 kinds of immune cells among subgroups, and selected differentially-expressed immune cells (*p* < 0.05) to analyze their influence on OS, after using the survminer package of R to calculate the optimal cutoff value of the corresponding immune cells for dividing tumor samples into two groups depending on the degree of infiltration. To further clarify immunological and molecular functions among subgroups, we performed single sample GSEA (ssGSEA) by using the GSVA package of R, and compared normalized ssGSEA scores among subgroups. Survival analysis was conducted for items with statistical differences (*p* < 0.05) according to the optimal cutoff value, which also calculated by the survminer package of R, of the items for dividing tumor samples into two groups. We evaluated the percentages of the three scores in immunized subtypes and final survival states. By using the time ROC package of R, we performed time-dependent receiver operating characteristic (ROC) curve analyses to obtain the area under the curve (AUC) and compared the prognostic value among composite score, m^6^A risk score, IRGPI, TIS, TIDE, interferon gamma (IFNG), MSI, Merck18, CD274, CD8, Dysfunction, Exclusion, myeloid-derived suppressor cells (MDSC), tumor-associated fibroblast (CAF), and TAM (M2) scores. The TIS score was calculated as the mean of normalized expression on a log_2_-scale of 18 characteristic genes (Ayers et al., 2017). Other scores were obtained online from the TIDE database (http://tide.dfci.harvard.edu/).

### Statistical analysis

The correlation among variables involved in the study was elucidated by Pearson correlation analysis. An independent *t*-test was performed to compare continuous variables among subgroups. Categorical data was tested by using the chi-square test. The Wilcoxon test was used to compare differences of TIS and scores obtained from TIDE database between subgroups. Multivariate survival analysis was performed using the Cox regression model, while other survival analyses were performed by Kaplan-Meier survival analysis with the log-rank test by using the survival package of R, and *p* < 0.05 was considered to indicate statistical significance. All the above analysis processes were shown in Supplementary Figure S1. All data was processed in SPSS (v.25.0) and R (v.4.1.2) software.

## Results

### Outcomes of differential expression analysis

In differential expression analysis of 454 HNSCC patient samples (43 normal vs. 454 tumor samples), 4,855 DEGs were identified, which included 3,550 upregulated genes and 1,305 downregulated genes (Supplementary Figures S2A, B), among which 495 upregulated and 143 downregulated were immune-related DEGs (Supplementary Figures S2C, D). Furthermore, a total of 312 differentially-expressed miRNAs were observed, of which 186 were upregulated and 126 were downregulated (Supplementary Figures S2E, F). Enrichment analysis showed that the 638 immune-related DEGs were associated with 1,995 GO items and 115 KEGG pathways (*q* < 0.05; *p* < 0.05, Supplementary Tables S1, 2; Supplementary Figures S2G, H).

### Immune-related hub genes and the IRGPI

**FIGURE 1:**
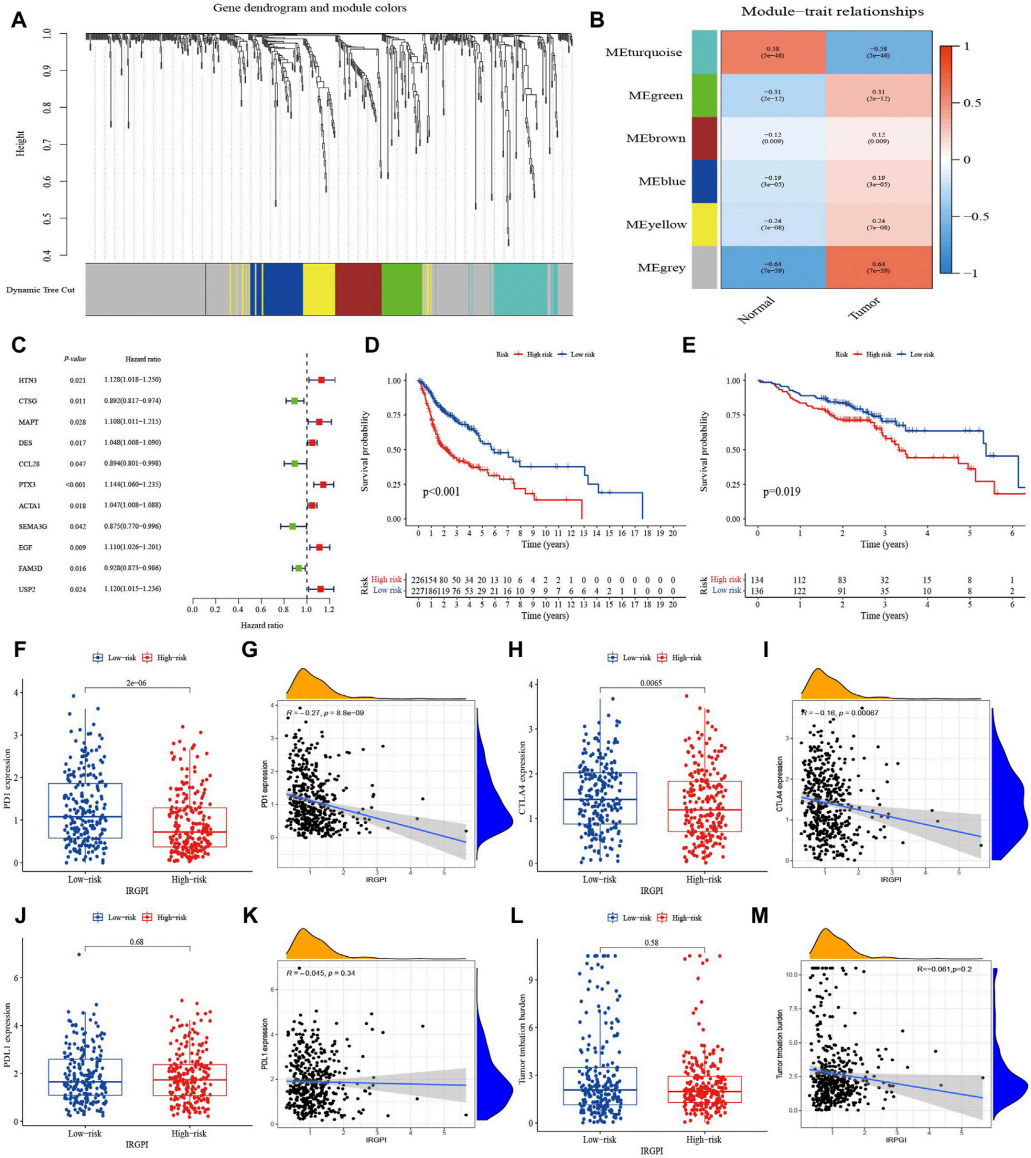
Identification of immune-related hub genes and comprehensive assessment of IRGPI. **(A)** Weighted gene coexpression network analysis (WGCNA) of immune-related differentially expressed genes with a soft threshold *β* = 7. **(B)** Gene modules related to HNSCC obtained by WGCNA. **(C)** Univariate Cox analysis of 11 immune-related hub genes. **(D)** K–M survival analysis of the IRGPI subgroups in TCGA cohort. **(E)** K–M survival analysis of the IRGPI subgroups in the GEO cohort. **(F–M)** The difference of PD-1, PD-L1, CTLA-4 and TMB in IRGPI subgroups (Wilcoxon test) and their correlation analysis with IRGPI.

To obtain immune-related hub genes, 498 samples were clustered to detect outliers (cutoff value 36,000) based on the 638 immune-related DEGs matrix, and the data was analyzed using WGCNA after deleting two outlier samples (Supplementary Figures S3A, B). The logarithm of nodes with connectivity of K (log(K)) was negatively correlated with the logarithm log[P(K)] of node probability, and the correlation coefficient was greater than 0.85. Based on a scale-free network, the optimal soft threshold power was 7 (Supplementary Figures S3C, D). The 638 immune-related DEGs were allocated to six modules, which were determined based on average linkage hierarchical clustering and optimal soft threshold power (Supplementary Figures S3E, F). According to the gene dendrogram and the Pearson correlation coefficient between module and sample feature for each module, the turquoise module, which was closely related to HNSCC and relatively concentrated genes distribution, was selected for further study (Figures 1A, B). There were 34 nodes and 115 edges of the turquoise module of the networks with a threshold weight >0.2 (Supplementary Figure S3G). Survival analysis showed that the expression of the 11 hub genes was closely related to the OS of HNSCC patients (*p* < 0.05, Supplementary Figures S4A–K). Turquoise module DEGs were analyzed by univariate Cox regression analysis, and 11 genes with statistical significance were selected as immune-related hub genes (*p* < 0.05, Figure 1C). We explored somatic mutations in the 11 hub genes and their clinicopathological information (Supplementary Table S3). Most of the 11 hub genes had missense mutations and non-sense mutations, but only the mutation rates of MAPT, EGF, PTX3, and SEMA3G were greater than 1% (Supplementary Figure S4L).

Multivariate Cox regression analysis on the 11 hub genes identified and provided coefficients for seven independent prognostic hub genes (HTN3, CTSG, MAPT, CCL28, PTX3, SEMA3G, and USP2). A prognostic index for tumor samples could be calculated by the formula: IRGPI = (HTN3 expression * 0.096) + (CTSG expression * −0.152) + (MAPT expression * 0.122) + (CCL28 expression * −0.094) + (PTX3 expression * 0.138) + (SEMA3G expression * −0.140) + (USP2 expression * 0.107). With the median IRGPI as the threshold, patients with high IRGPI had worse OS than those with low IRGPI (*p* < 0.001, Figure 1D). This result was verified in the HNSCC dataset of GSE65858 (*n* = 270) (*p* < 0.05, Figure 1E).

Expression of PD-1 and CTLA-4 were high in the IRGPI-low subgroup and correlation analysis indicated slightly negatively correlated with the IRGPI (Figures 1F–I). However, TMB and expression of PD-L1 did not show significant differences and relationship with IRGPI (Figures 1J–M).

### Landscape and risk score of m^6^A regulators in HNSCC

**FIGURE 2:**
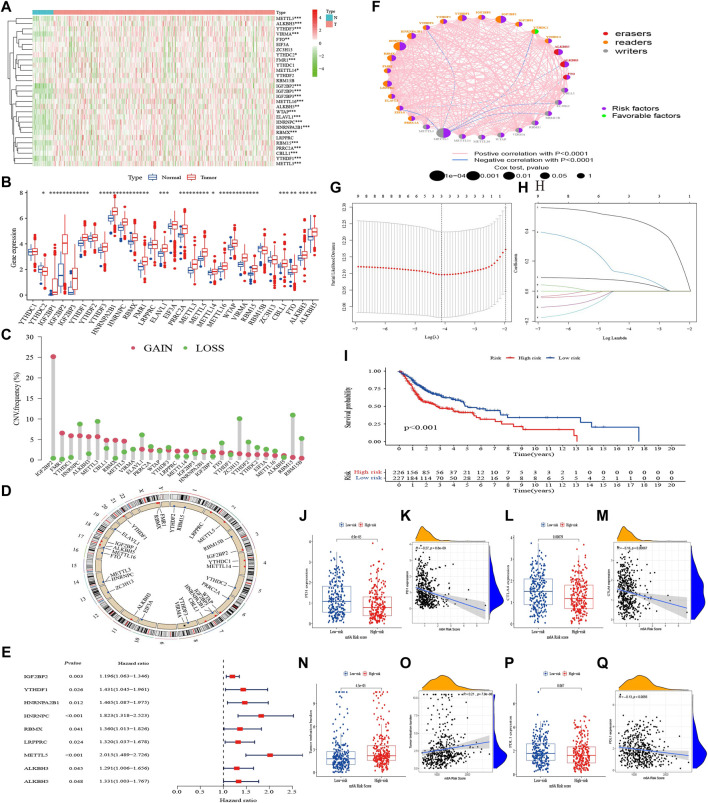
Landscape of m^6^A regulators and m^6^A risk score in HNSCC. **(A)** Heatmap of 29 m^6^A regulators in TCGA cohort. **(B)** The upper and lower ends of the boxes represented interquartile range of values. The lines in the boxes represented median value, and dots showed outliers. The asterisks represented the statistical *p*-value (*p < 0.05; **p < 0.01; ***p < 0.001). **(C)** The CNV variation frequency of m^6^A regulators. The height of the column represented the alteration frequency. **(D)** The location of CNV alteration of m^6^A regulators on 23 chromosomes. **(E)** Univariate Cox analysis of 29 m^6^A regulators. **(F)** The interaction between m6A regulators in HNSCC. **(G, H)** Determination of the m6A regulators strongly correlated with prognosis by the LASSO analysis. **(I)** K–M survival analysis of the m^6^A risk score subgroups in TCGA cohort. **(J–Q)** The difference of PD-1, PD-L1, CTLA-4, and TMB in m^6^A risk score subgroups (Wilcoxon test) and their correlation analysis with m^6^A risk score.

There were 29 m^6^A regulators in this study, including 10 writers, three erasers, and 16 readers. As shown in Figures 2A, B, 23 of them, including 12 readers, 8 writers, and all erasers, were differentially expressed between HNSCC tumor and para-cancer samples, and were upregulated in tumor samples except for the reader YTHDC2. Based on gene expression of regulators, we analyzed the correlation between 29 m^6^A regulators in pairs with a *p* < 0.001 signifying a correlating pair. The results showed that m^6^A regulators were generally positively correlated, but only the reader YTHDF3 and PRRC2A were highly correlated (*r* = 0.77, Supplementary Figure S5A).

We also explored mutations and clinicopathological information of the m^6^A regulators (Supplementary Figure S5B). In 451 HNSCC samples, m^6^A regulator mutations occurred in 68 cases, with a mutation frequency of 15.08%. The results showed that readers PRRC2A and YTHDC2 had the highest mutation frequency (about 2%) among the 29 regulators. The CNV frequency showed it was very common among regulators, with 15 undergoing copy number amplification, and 14 undergoing copy number deletion (Figure 2C). The chromosomal locations of m^6^A regulators CNV were shown in Figure 2D.

Univariate Cox regression demonstrated that IGF2BP2, YTHDF1, HNRNPA2B1, HNRNPC, RBMX, LRPPRC, METTL5, ALKBH3, and ALKBH5 were correlated with OS (Supplementary Figure S6), with hazard ratios all greater than 1 (*p* < 0.05, Figure 2E). Based on the results of univariate Cox regression analysis, we constructed an m^6^A regulator network (Figure 2F), which showed the comprehensive landscape of the correlation of regulators in pairs and their prognostic significance for HNSCC. We found that all regulators except YTHDC2 were prognostic risk factors, and there was a widespread positive correlation between the expression of regulators. The LASSO algorithm was used to obtain the coefficient of regulators with prognostic value (Figures 2G, H), IGF2BP2, HNRNPC, and METTL5 were selected to construct a prognostic signature using the cohort in TCGA. The formula was as follows: m^6^A risk score = (0.067 * IGF2BP2 expression) + (0.119 * HNRNPC expression) + (0.487 * METTL5 expression). According to the median risk score, HNSCC patients with a low m^6^A risk score had better OS than those with a high one (*p* < 0.001, Figure 2I).

The expression of PD-1 and CTLA-4 were high and TMB was low in the m^6^A-risk-low subgroup and correlation analysis indicated slightly positively correlated with m^6^A risk score (Figures 2J–O). Expression of PD-L1 did not show significant differences, but negative correlated with the score (Figures 2P, Q).

### Classification and composite score

**FIGURE 3:**
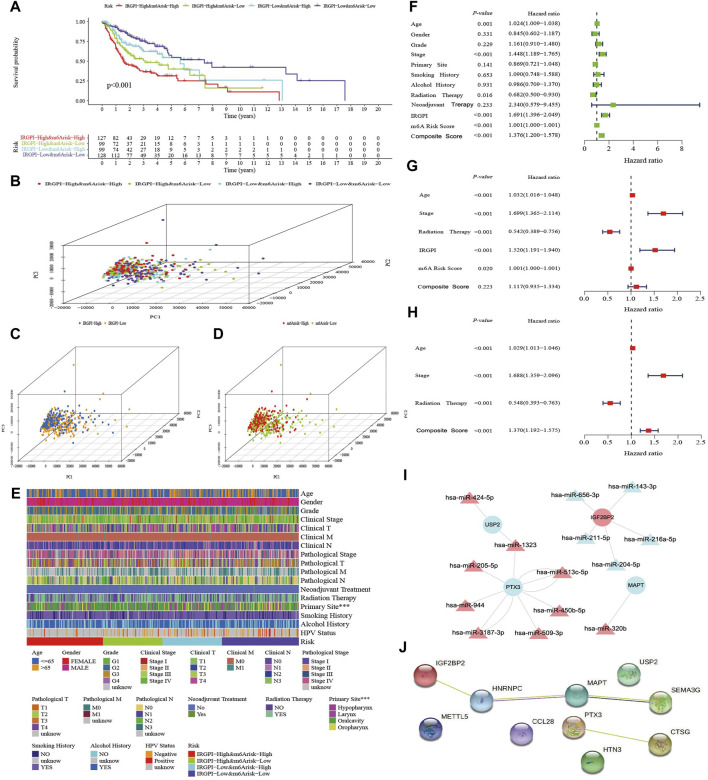
Assessment of the composite score. **(A)** K–M survival analysis of the composite score subgroups in TCGA cohort. **(B–D)** Principal component analysis of the transcriptome profiles of the three score-defined subgroups. **(E)** Heatmap of clinical relevance for composite score subgroups (***p < 0.001). **(F)** Univariate Cox analysis of clinicopathologic factors, IRGPI, m^6^A risk score and the composite score. **(G)** Multivariate Cox analysis of the factors significant in the univariate Cox analysis (*p* < 0.05). **(H)** Multivariate Cox analysis of composite score and clinicopathologic factors significant in the univariate Cox analysis (*p* < 0.05). **(I)** The target interaction network of differentially expressed miRNA and the 10 mRNAs which are expressed by the genes used to construct the three scores. **(J)** The Protein-Protein Interaction (PPI) Network of 10 genes used to construct the three scores.

Based on the IRGPI and m^6^A risk score, HNSCC patients in TCGA were pairwise combined as subgroup A (IRGPI-High&m^6^A-risk-High, *n* = 127), subgroup B (IRGPI-High&m^6^A-risk-Low, *n* = 99), subgroup C (IRGPI-Low&m^6^A-risk-High, *n* = 99) and subgroup D (IRGPI-Low&m^6^A-risk-Low, *n* = 128). IRGPI and m^6^A risk score were standardized and multiplied by the weight coefficient obtained from PCA to obtain the composite score, using the formula: Composite score = 0.5*Z-score (IRGPI) + 0.5*Z-score (m^6^A risk score). Survival analysis showed from subgroup A to D, the OS was better than that of the previous subgroup (*p* < 0.001, Figure 3A). The PCA based on subgroup gene expressions is shown in Figures 3B–D. The correlation of the composite score and clinicopathological information in Figure 3E showed the correlation between the composite score and the primary site was statistically significant (*p* < 0.001). Univariate Cox regression analysis showed that age, stage, radiotherapy, IRGPI, m^6^A risk score and composite score were significantly correlated with the prognosis of HNSCC (Figure 3F). Multivariate Cox regression analysis confirmed that the IRGPI and m^6^A risk score were independent prognostic factors after adjusting for other clinicopathological factors (Figure 3G), and the composite score was also an independent prognostic factor after excluding the effects of IRGPI and m^6^A risk score (Figure 3H). Combining the 10 genes, used to construct composite score, with differentially-expressed miRNA to construct a gene targeted network with 24 nodes and 18 edges showed that miRNA was mainly upregulated to inhibit gene expression in the network (Figure 3I). The PPI network revealed gene fusion between HNRNPC and MAPT, adjacent of MAPT and SEMA3G, and other interrelationships obtained from text mining (Figure 3J; Supplementary Table S4).

### Molecular characteristics of different subgroups

GSEA was performed to determine the GO, KEGG and Hallmark gene sets enriched in the different subgroups (*p* < 0.05, Supplementary Table S5). The gene sets of subgroup A were markedly enriched in tumor metastasis and drug resistance (Supplementary Figure S7A), the gene sets of subgroup B were markedly enriched in tumor growth promotion and immune rejection (Supplementary Figure S7B), the gene sets of subgroup C were markedly enriched in immune rejection and DNA repair (Supplementary Figure S7C), and the gene sets of subgroup D were markedly enriched in immune response (Supplementary Figure S7D).

**FIGURE 4:**
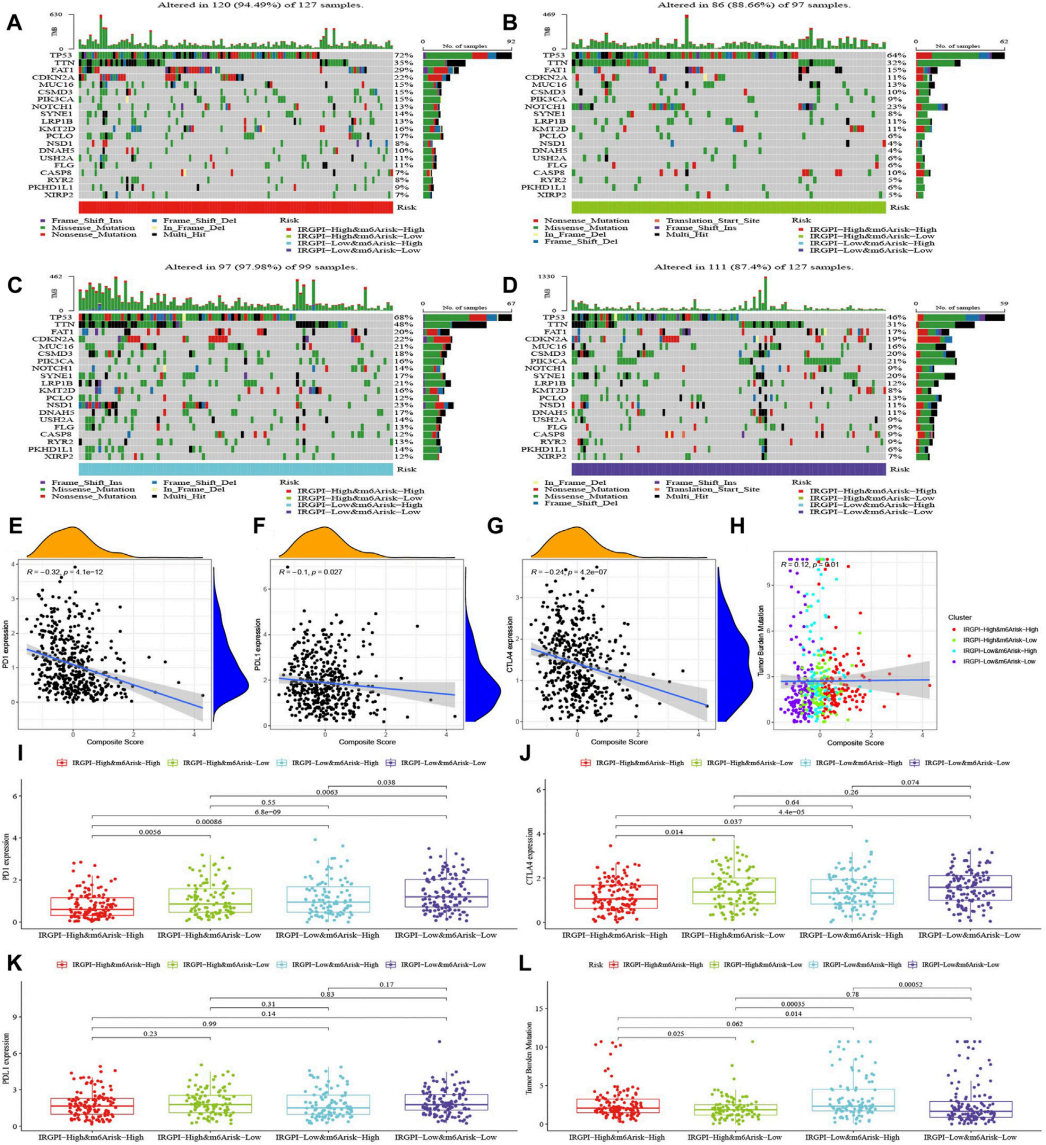
Characteristics of tumor somatic mutations, PD-1, PD-L1, CTLA-4, and TMB in composite subgroups. **(A–D)** Mutation profiles of the 20 genes with the highest mutation rates in HNSCC samples of the TCGA cohort in the four composite score subgroups. **(E–H)** The correlation analysis of PD-1, PD-L1, CTLA-4, and TMB with the composite score. **(I–L)** The difference of PD-1, PD-L1, CTLA-4, and TMB in composite score subgroups (Wilcoxon test).

The top 20 highest mutation rate genes in TCGA cohort demonstrated that the most common mutation type was missense mutation, followed by non-sense mutation and frameshift deletion, as plotted in Figures 4A–D. The mutation frequencies of TP53, TTN, and FAT1 were all greater than 15% among the four subgroups, CDKN2A, MUC16, CSMD3, and PIK3CA were greater than 15% in subgroups A, C, and D, KMT2D was greater than 15% in subgroups A and C, SYNE1 was greater than 15% in subgroups C and D, PCLO was greater than 15% in subgroup A, NOTCH1 was greater than 15% in subgroup B, and LRP1B, NSD1, and DNAH5 were greater than 15% in subgroup C.

We discovered that the composite score was moderately negative correlated with PD-1, slightly negative correlated with CTLA-4 and PD-L1, and slightly positive correlated with TMB (Figures 4E–H), and their differences across subgroups were shown in Figures 4I–L.

### Immune characteristics of different subgroups

**FIGURE 5:**
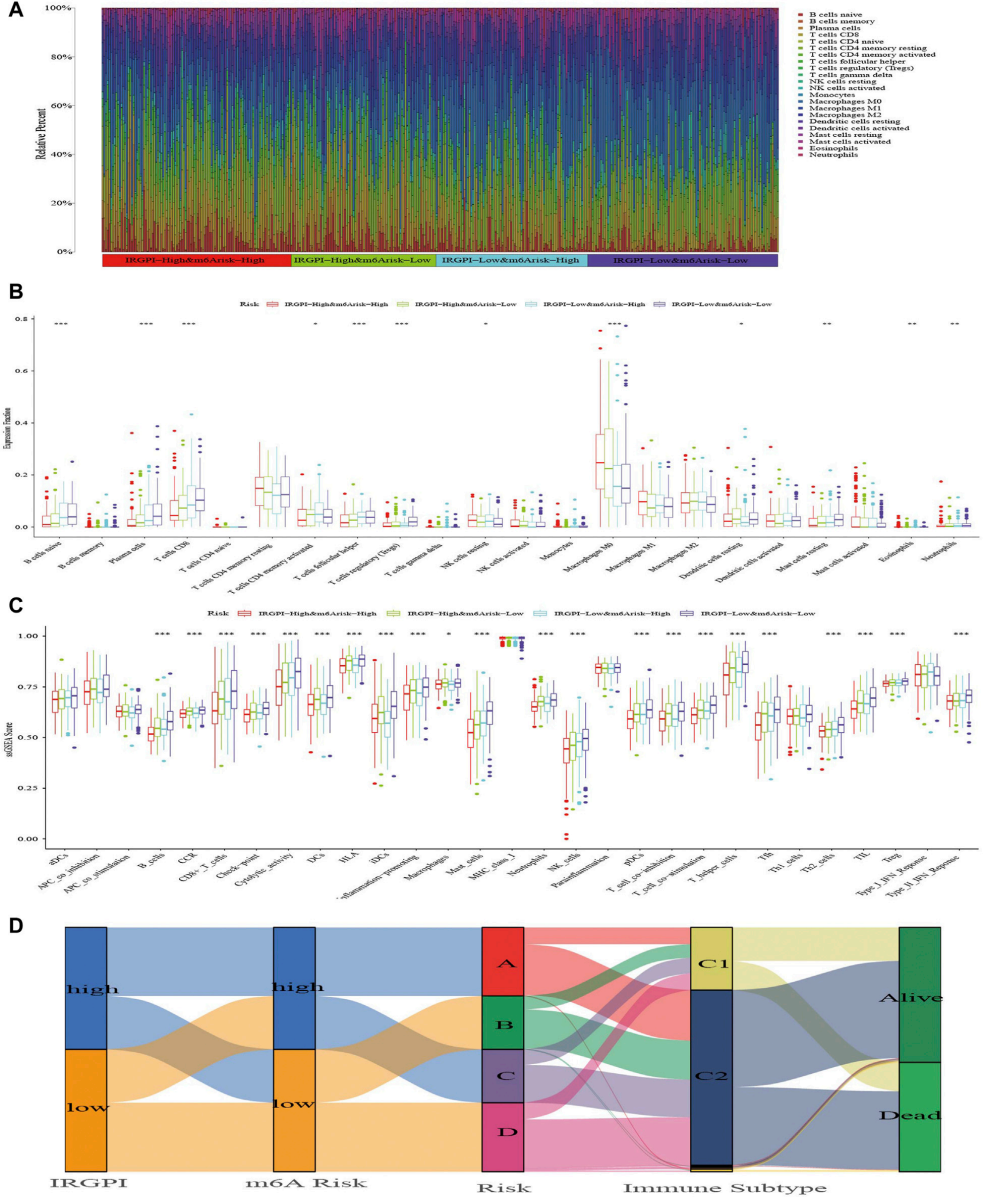
The TIME landscape and the immune subtype of different composite subgroups. **(A)** The proportions of TIME cells in composite score subgroups for patients in TCGA cohort. **(B)** The abundance of each TIME infiltrating cell in composite score subgroups. The upper and lower ends of the boxes represented interquartile range of values. The lines in the boxes represented median value, and dots showed outliers. The asterisks represented the statistical *p*-value (*p < 0.05; **p < 0.01; ***p < 0.001). **(C)** The molecular and immune-related function of composite score subgroups. The gene sets of molecular and immune-related function were analyzed by the single simple gene set enrichment analysis (ssGSEA) and then compared between different composite score subgroups. The upper and lower ends of the boxes represented interquartile range of values. The lines in the boxes represented median value, and dots showed outliers. The asterisks represented the statistical *p*-value (*p < 0.05; **p < 0.01; ***p < 0.001). **(D)** The Alluvial diagram shows changes in IRGPI, m^6^A risk score, composite score, immune subtype, and survival status of patients in the TCGA cohort.

The abundance of 22 types of immune cells in different subgroups was analyzed with the Wilcoxon test to compare their distribution (Supplementary Table S6). The abundance of native B cells, plasma cells, CD8^+^ T cells, activated memory CD4^+^ T cells, follicular helper T cells, regulatory T cells (Tregs), resting natural killer (NK) cells, M0 macrophages, resting dendritic cells, resting mast cells, eosinophils and neutrophils were significantly different in subgroups (*p* < 0.05, Figures 5A,B). The abundance of M0 macrophages was the highest in all subgroups, and the patients with higher abundance of M0 macrophages had lower OS. In contrast, patients with the high abundance of native B-cell, plasma cells, CD8^+^ T cells, follicular helper T cells and Tregs had better OS. Based on the optimal cutoff value of the abundance of each immune cell, HNSCC patients were divided into two groups for survival analysis, which indicated that the more abundance of follicular helper T cells, Tregs, activated memory CD4^+^ T cells, CD8^+^ T cells, activated B cells, plasma cells, resting dendritic cells and resting mast cells, the better the OS. In contrast, a high abundance of neutrophils, activated mast cells, and M0 and M2 macrophages tended to imply poor OS (Supplementary Figure S8).

Next, we applied specific gene signatures by ssGSEA to define immunity and molecular function among the subgroups (Supplementary Table S7). As the results illustrate, the better the OS of the subgroup, the more cytolytic activity, mast cells and NK cells (Figure 5C). Also, a variety of T cells and plasma cells, immune suppression and signals related to tumor metastasis had statistical significance, which were more obvious in subgroups A and D, and these immune signatures occurred most commonly in subgroup D with the best OS. Based on the optimal cutoff value of each immune and molecular function signature for the tumor samples in TCGA, it revealed the signatures had a large impact on survival. Patients with more macrophages and parainflammatory cells had poor prognoses, while patients with more T cells, mast cells, NK cells, B cells and other signatures has better prognosis (Supplementary Figure S9).

### The benefit of immunotherapy in different subgroups

Thorsson et al. (2018) divided all TCGA tumor samples into six immune subtypes, which included wound healing (Immune C1), IFN-gamma dominant (Immune C2), inflammatory (Immune C3), lymphocyte-depleted (Immune C4), immunologically quiet (Immune C5) and TGF-beta-dominant (Immune C6), and showed patients who belonged to types C1, C2 and C3 could benefit from neoadjuvant immunotherapy following surgery. We explored the proportion of HNSCC samples in TCGA cohort and found that almost all samples belonged to the C1 and C2 immune subtypes. In each subgroup, C2 was predominant with similar proportion of C1 and C2 (Figure 5D; Supplementary Table S8).

**FIGURE 6:**
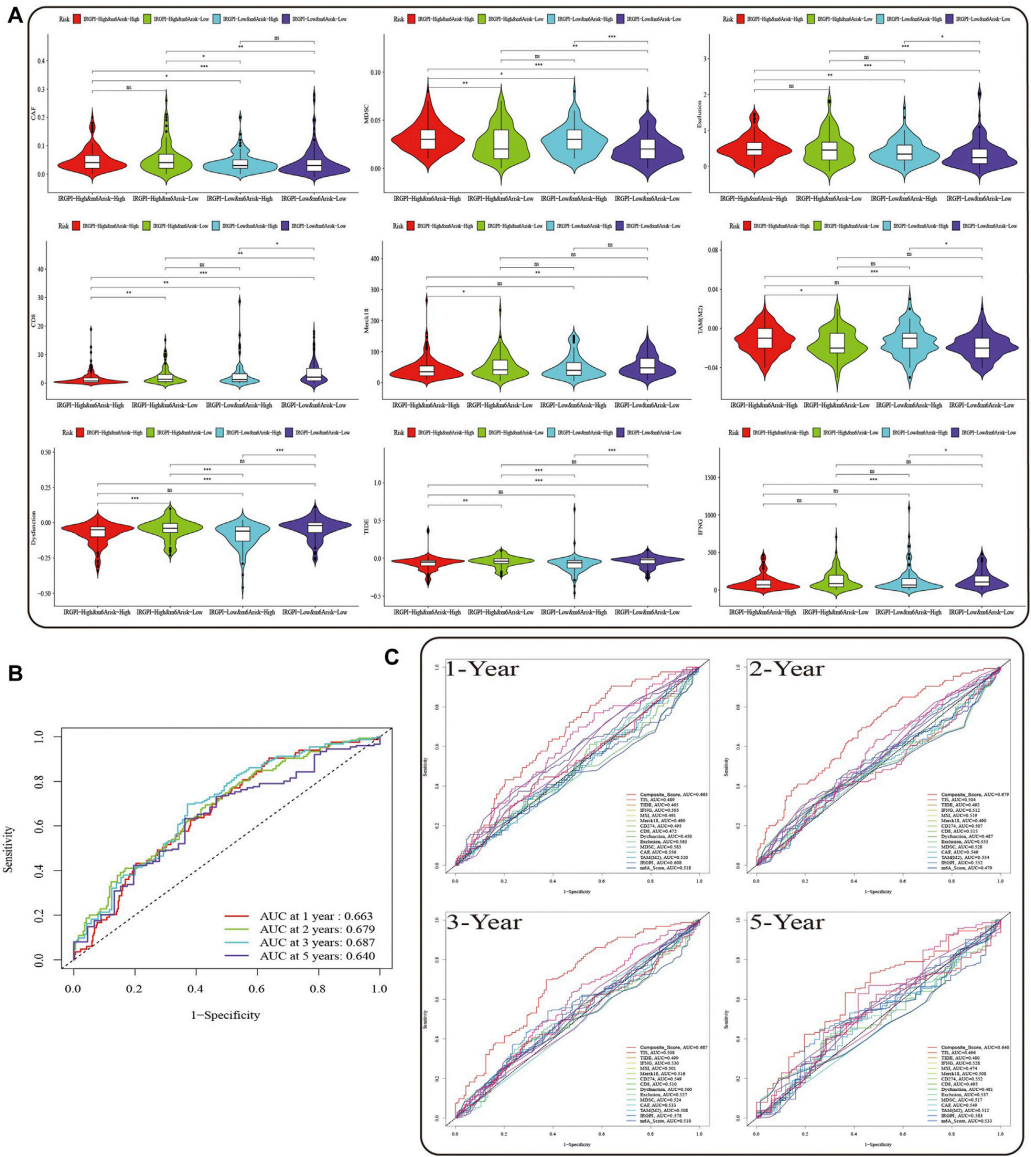
Prognostic value of the composite score in the TCGA cohort. **(A)** TIDE, Dysfunction, Exclusion, TAM (M2), IFNG, CD8, CAF, MDSC, and Merck18 scores in different composite score subgroups. The scores between the two subgroups were compared through the Wilcoxon test (ns, not significant, *p < 0.05; **p < 0.0s1; ***p < 0.001). **(B)** The predictive value of composite score in TCGA HNSCC cohort at 1, 2, 3, and 5 years. **(C)** ROC analysis of composite score, IRGPI, m^6^A risk score and the scores of TIS, TIDE, IFNG, MSI, Merck18, CD274, and CD8, Dysfunction, Exclusion, MDSC, CAF, and TAM (M2) on OS at 1-, 2-, three- and 5-year follow-up in TCGA HNSCC cohort.

We used TIDE, IFNG, MSI, Merck18, CD274, CD8, Dysfunction, Exclusion, MDSC, CAF, and TAM (M2) scores to evaluate the potential clinical efficacy of immunotherapy in subgroups categorized by IRGPI, m^6^A risk score and composite score. In the IRGPI-high subgroup, TAM (M2), Exclusion, CAF and MDSC scores were higher, while CD8 and IFNG scores were higher in the IRGPI-low subgroup (Supplementary Figure S10A). TAM (M2), Exclusion and MDSC scores were higher in the m^6^A risk-high subgroup, while TIDE, CD8, Dysfunction, IFNG, and Merck18 scores were higher in the m^6^A risk-low subgroup (Supplementary Figure S10B). There were no statistically significant differences in the IRGPI and m^6^A risk score subgroups for the remaining scores. In the subgroups of composite score, the scores of CD274 and MSI did not differ among subgroups, while TIDE, Dysfunction, Exclusion, TAM (M2), IFNG, CD8, CAF, MDSC, and Merck18 scores demonstrated significant differences between subgroup A and subgroup D, but their differences among other subgroups were not uniform (Figure 6A). In general, patients in subgroup A may have the best benefit from immunotherapy, while patients in subgroups B and C had better immunotherapy benefit than patients in subgroup D. However, the magnitude of immunotherapy benefit between subgroups B and C remains to be further delineated.

Subsequently, we evaluated the OS of the composite score and other prognostic indicators at 1, 2, 3, and 5 years by ROC curves, and found that prediction of OS within 3 years by the composite score was better than the long-term survival rate (Figure 6B, AUC = 0.663 (1-year), AUC = 0.679 (2-year), AUC = 0.687 (3-year), AUC = 0.640 (5-year)). Moreover, the predictive value of composite score for OS was superior to any other scores (Figure 6C).

## Discussion

Increasing evidence indicates that multiple m^6^A regulators perform a vital role in regulating the occurrence, development, metastasis, drug resistance, radiotherapy resistance and other aspects of HNSCC (Liu et al., 2020; Jing et al., 2021; Wu et al., 2021; Jin et al., 2022; Yu et al., 2022). At the same time, in-depth research has uncovered more and more m^6^A regulators. Currently, characteristics of the HNSCC TIME mediated by the combined action of multiple m^6^A regulators have not been sufficiently explained (Jing et al., 2021). On the other hand, immunotherapy is widely applied as an effective method to treat HNSCC, but the overall response rate remains low. After years of exploring HNSCC prognostic signatures, while some simple signatures can forecast immunotherapy and OS (Fasano et al., 2022; Poulose and Kainickal, 2022), single prognostic signatures cannot very well predict which patients can benefit from immunotherapy. Compound signatures constructed by combining multiple signatures can further differentiate the prognosis and immune response of patients with diverse OS, which will contribute to our understanding of anti-tumor immune response of TIME and guide more effective immunotherapy strategies. It is inevitable that future research will focus on identifying compound immunotherapy prognosis signatures.

Here, we used different methods to establish three different prognostic scores, all of which are valid independent prognostic signatures for HNSCC. Compared with IRGPI and m^6^A risk score, the composite score is more detailed for OS stratification of HNSCC patients, and the AUC value is greater than other scores, which means that the prediction of prognosis is also better.

In this research, based on 29 m^6^A regulators, we reveal the m^6^A methylation landscape of HNSCC in more detail. We revealed that mutation rate of the 29 m^6^A regulators was not significant. Interestingly, about half of them showed increasing of CNV which might indicated that m^6^A regulators did not affect HNSCC primarily through mutation and CNV. Except for YTHDC2, all regulators were highly expressed in HNSCC samples and were prognostic risk factors. However, publications on m^6^A regulators in HNSCC are limited, and further studies are warranted to clarify the effect of m^6^A on HNSCC.

On the basis of the composite score, we further studied gene mutations of subgroups. The greatest differences in gene mutations among subgroups were TP53 mutations, which were more frequent in subgroup A than in subgroup D (72% vs. 46%). TP53 mutation is not only the most common genetic event in cancer, but is also associated with more aggressive disease and poorer patient outcomes in many cancers, especially in HNSCC (Poeta et al., 2007; Leroy et al., 2014). The Notch signaling pathway is known to be an evolutionarily conserved intercellular signaling pathway, and NOTCH1 mutation is associated with HNSCC (Schmidl et al., 2022; Zhou et al., 2022). The mutation rate of NOTCH1 gene in subgroup B (23%) is higher than that in other groups, which may be the reason why HNSCC patients in subgroup B have worse OS than those in subgroup C. It also means that some HNSCC patients may benefit from anti-NOTCH1 therapy. The TTN mutation rate of subgroup C (48%) is higher than that of other groups, and is mainly comprised of multiple mutations, but its prognosis and biological significance of HNSCC is unknown. Subsequently, we explored the relationship between three scores and known immunotherapy predictive signatures PD-1, PD-L1, CTLA-4, and TMB, which was evaluated in prospective clinical trials as a potential signature for predicting immune checkpoint inhibitor therapy response in HNSCC (Cristescu et al., 2018; Chan et al., 2019). In addition, a number of clinical trials have made breakthroughs in immunotherapy using immune checkpoint inhibitors against HNSCC, and a number of combined anti-CTLA-4 and anti-PD-1/L1 clinical trials are also underway (Ferris et al., 2020; Maio et al., 2021; Masarwy et al., 2021). The expression of PD-1 and CTLA-4 was synochronous in each composite score subgroups, which might indicate that combination of anti-CTLA-4 and anti-PD-1 therapy would increase the anti-cancer effects, especially in subgroup D.

Understanding the TIME landscape of HNSCC can help discover new treatments or alter the TIME to improve the effectiveness of immunotherapy (Feng and Hess, 2021; Watermann et al., 2021). The immunotyping by Thorsson et al. (2018) revealed that almost all HNSCC patients should receive neoadjuvant immunotherapy. As we know, immune-excluded and immune-desert phenotypes can be considered non-inflammatory tumors (Bagaev et al., 2021). Subgroup A corresponding to the immune-desert phenotype is characterized by immunosuppression, subgroups B and C corresponding to the immune-excluded phenotype are characterized by the activation of innate immunity and stroma, and subgroup D corresponding to the immune-inflamed phenotype is characterized by activation of adaptive immunity, known as a hot tumor (31), which is characterized by a large infiltration of immune cells. After exploring the characteristics of immune cell infiltration in the TIME of different subgroups, although the degree of immune infiltration in subgroup B was similar to that in subgroup C, low NK and mast cell infiltration and low cytolytic activity might be the reason for the worse prognosis of subgroup B compared to subgroup C (Schantz et al., 1986; Attramadal et al., 2016; Gu et al., 2019; Charap et al., 2020; Watermann et al., 2021). However, the tumor immune cell infiltration in subgroup A was the least, indicating a possible worse result of immunotherapy comparing to any other subgroup. In the TIME of HNSCC, infiltration of undifferentiated macrophages (M0) was the highest among the 22 types of immune cells, and there was a significant difference of the composite score between the subgroups. TAM (M2) is associated with elevated tumor growth, aggressive phenotype and poor prognosis in solid tumors (Komohara et al., 2016; Ngambenjawong et al., 2017; Chen et al., 2019). Although there was no statistical difference in the expression of TAM (M2) between the composite score subgroups, the infiltration by TAM (M2) was high and statistically significant in the high-risk IRGPI or m^6^A risk score subgroups, which is consistent with the results of our survival analysis. In addition, tumor drug resistance is the main cause of chemotherapy failure, and studies have shown that TAMs are closely related to the drug resistance of tumors (Lopez-Yrigoyen et al., 2021; Pu and Ji, 2022). CAR-M have been demonstrated to enter solid tumors and survive in the tumor environment, triggering long-lasting adaptive immune responses (Komohara et al., 2016; Sloas et al., 2021). Accordingly, the excessive TAM infiltration indicates that HNSCC patients are expected to benefit from anti-TAM and CAR-M therapy, while the patients of subgroup A corresponding to the immune desert type may benefit the most, which also means that HNSCC patients who fail other immunotherapies or chemotherapy may benefit. In addition, MDSCs are the precursors of dendritic cells, macrophages and granulocytes, and have the ability to inhibit the immune response (Draghiciu et al., 2015; Greene et al., 2020; Grover et al., 2021). The results of our study indicate that subgroup A patients, who have worse prognosis, also have a higher MDSC score. By evaluating the MDSC score of subgroups, defined by IRGPI, m^6^A risk score and composite score, we found that the composite score was more favorable in identifying the MDSC-related risks of HNSCC. This helps identify HNSCC patients who may benefit from anti-MDSC-targeted therapy. Evidently, compared with IRGPI and m^6^A risk scores, the composite score can provide a more precise grouping of HNSCC patients, which is conducive to a more detailed understanding of the TIME of HNSCC and guide more effective immunotherapy strategies.

Finally, we compared the predictive prognostic ability of composite score with IRGPI, m^6^A risk score, TIS, TIDE, IFNG, MSI, Merck18, CD274, CD8, Dysfunction, Exclusion, MDSC, CAF, and TAM (M2) scores on the HNSCC cohort in TCGA. The results showed that the composite score was better than other scores in predicting both short-term and long-term survival. Although the AUC value of composite score declined slightly at 5 years, this might due to the limited number of patients with a 5-year OS.

In conclusion, the composite score, constructed based on IRGPI and m^6^A risk score, is an independent prognostic signature, which is more useful in identifying immune and molecular characteristics, predicting patient prognosis and guiding therapy as compared with single prognostic signatures. Further studies are still needed to clarify that the composite score is potential prognostic indicator for immunotherapy.

## Data Availability

The original contributions presented in the study are included in the article/Supplementary Material, further inquiries can be directed to the corresponding author.
