# Supplementary material for: A composite score based on immune-related gene prognostic index and m6A risk score of head and neck squamous cell carcinoma
Source: Front Genet. 2023 Feb 9;14:1061569. doi: 10.3389/fgene.2023.1061569 (PMC9948032; doi:10.3389/fgene.2023.1061569)
Supplement: Supplementary file 2 [file Image1.PDF]

Figure S1. The main flow chart of this study.

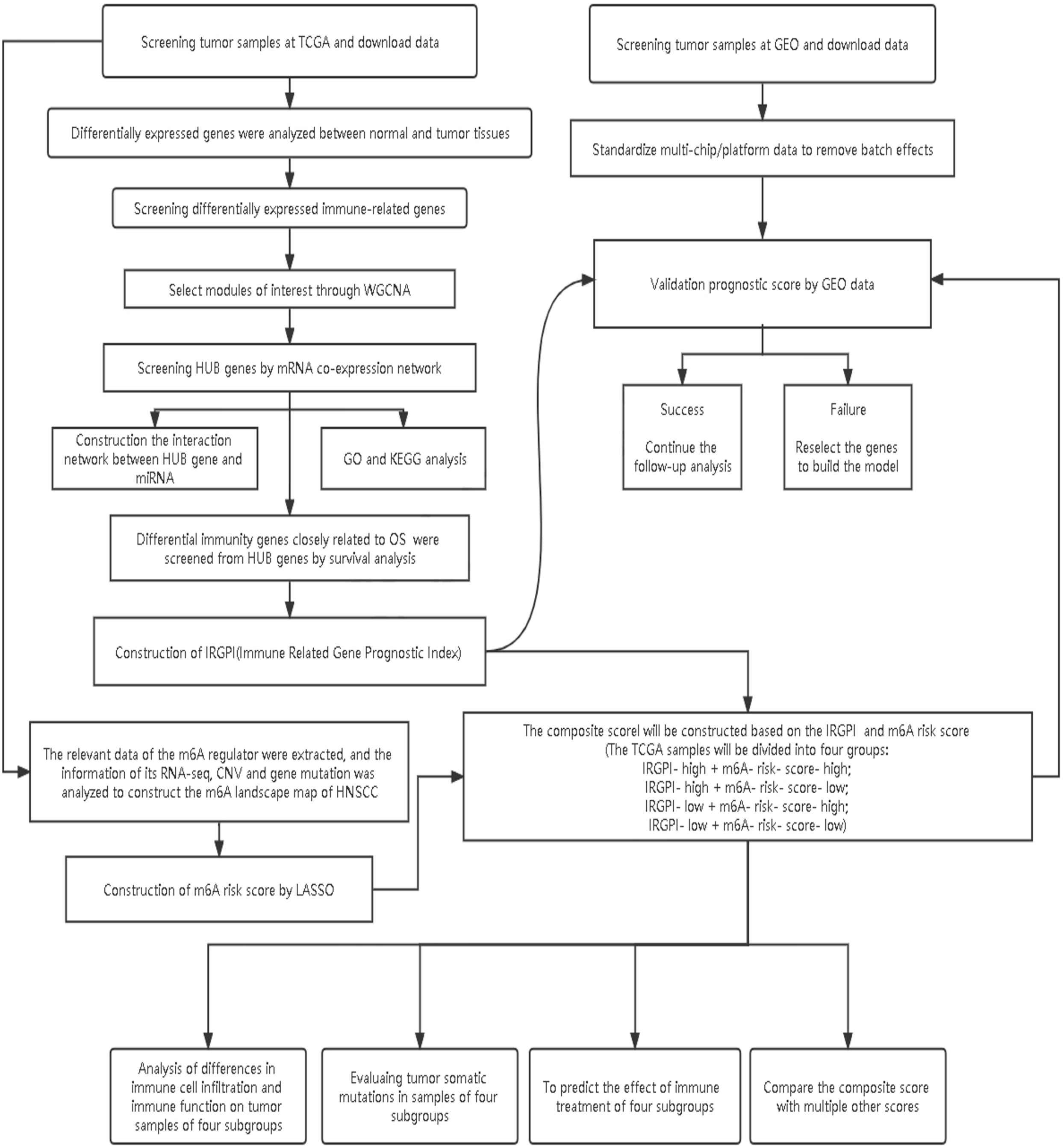

Figure S2. Outcomes of differential expression analysis and the enrichment analysis of GO and KEGG.

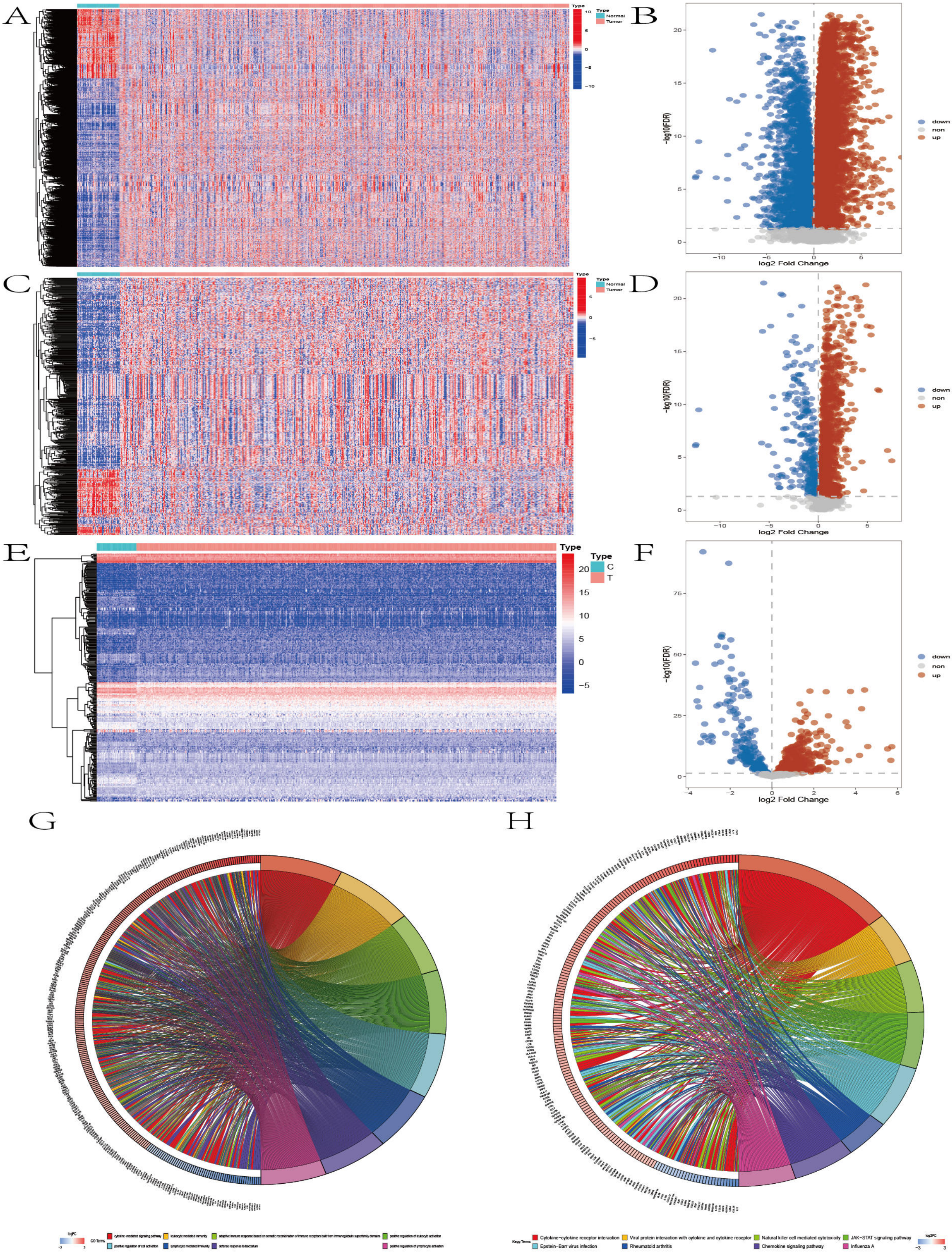

(A) Heatmap displaying all differentially expressed genes (DEGs) between 454 HNSCC samples (red) and 43 para-cancer samples (blue) (FDR < 0.05, |log2FC| > 1).
(B) Volcano plot of all DEGs, each point on the graph represents a gene , red: increased expression in tumor samples; blue: decreased expression in tumor samples; grey: no statistical difference.
(C) Heatmap displaying immune-related DEGs between 454 HNSCC samples (red) and 43 para-cancer samples (blue).
(D) Volcano plot of immune-related DEGs.
(E) Heatmap displaying all differentially expressed miRNAs between 454 HNSCC samples (red) and 43 para-cancer samples (blue) (FDR < 0 .05, |log2FC| > 1).
(F) Volcano plot of all differentially expressed miRNAs.
(G) Gene Ontology (GO) enrichment analysis of the immune-related DEGs ($q < 0.05$ & $p < 0.05$).
(H) Kyoto Encyclopedia of Genes and Genomes (KEGG) pathway analysis of the immune-related DEGs ($q < 0.05$ & $p < 0.05$).

Figure S3. Identification of immune-related hub genes.

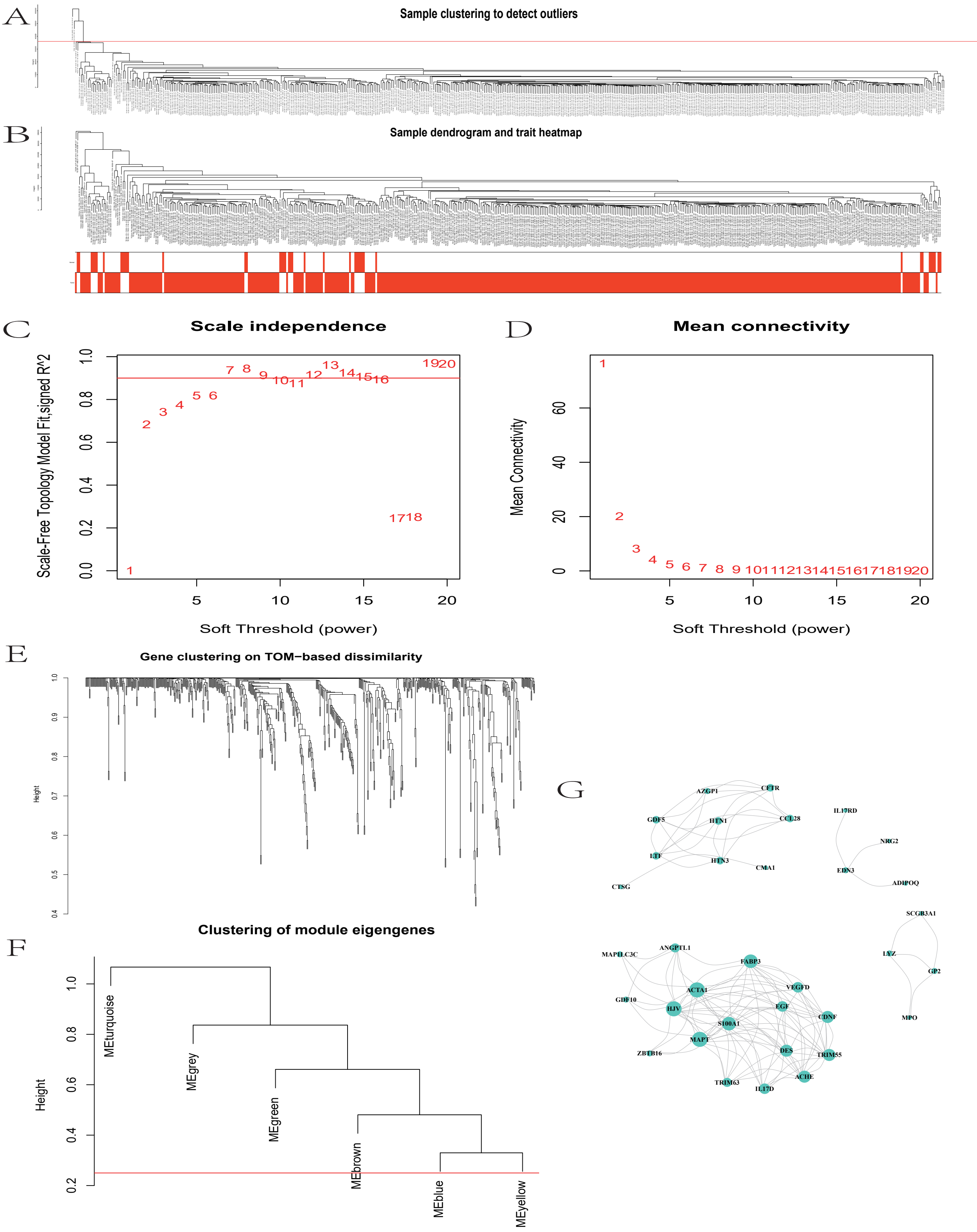

(A) Samples from the TCGA cohort were clustered to remove outlier samples, and the red line represents the cutoff value (36000).
(B) Sample dendrogram and trait heatmap of the TCGA cohort after removal of outlier samples.
(C-D) Determination of the soft-thresholding power in the WGCNA analysis. In the scale independence graph, the horizontal line indicates that the threshold value is 0.85. As seen from the two graph, the optimal soft threshold for WGCNA was 7.
(E) Gene clustering on TOM-based dissimilarity in the WGCNA analysis.
(F) Clustering of module eigengenes to identify six modules by setting the merging threshold function at 0.25.
(G) Gene co-expression network for genes with edge weights greater than 0.2 in the turquoise module.

Figure S4. Kaplan-Meier curves and alteration events of 11 immune-related hub genes.

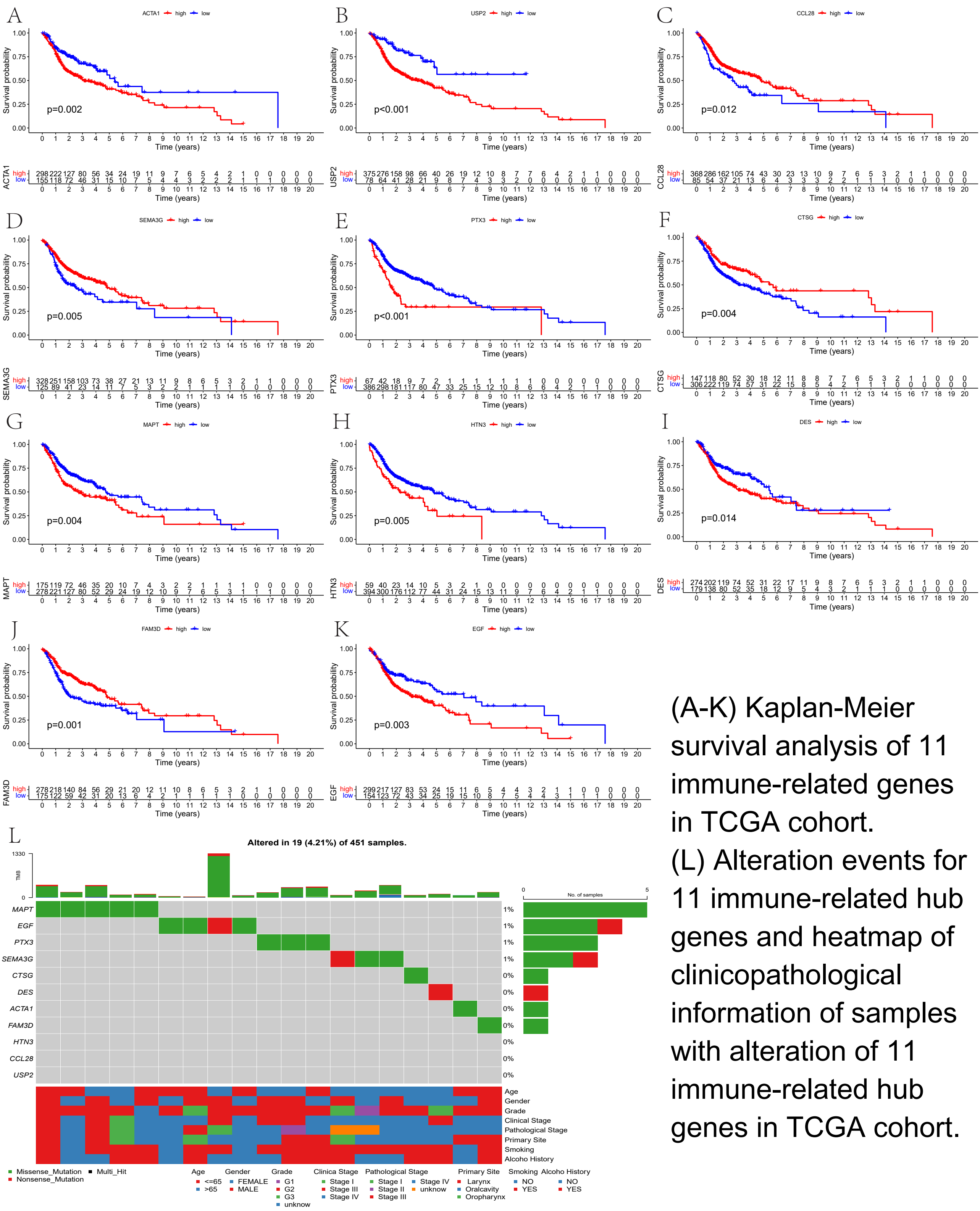

(A-K) Kaplan-Meier survival analysis of 11 immune-related genes in TCGA cohort.

(L) Alteration events for 11 immune-related hub genes and heatmap of clinicopathological information of samples with alteration of 11 immune-related hub genes in TCGA cohort.

Figure S5. Relationships and mutations of m6A regulators.

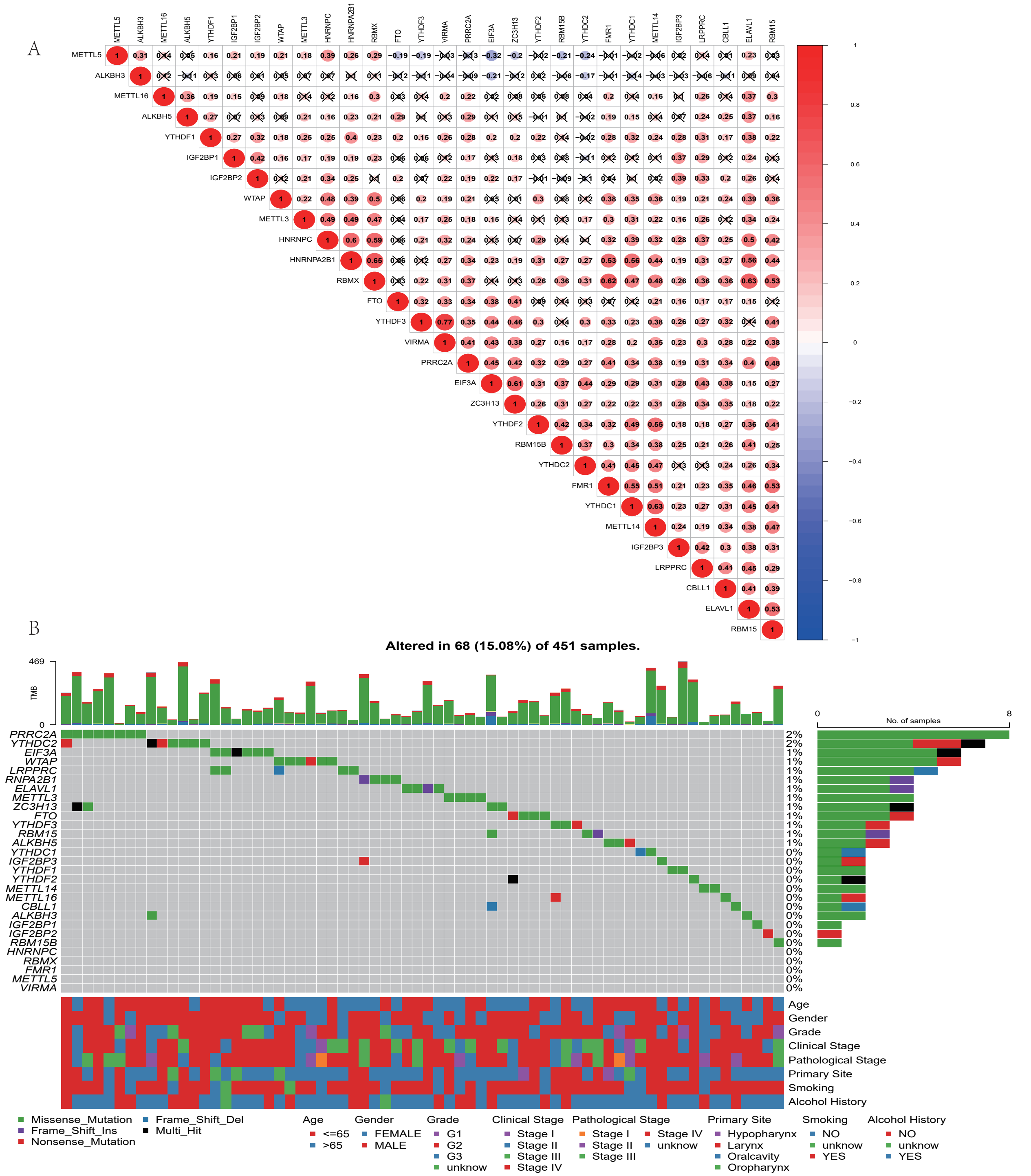

(A) Correlation analysis of 29 m6A regulators based on gene expression, numbers on the graph represent correlation coefficients, red represents positive correlation, blue represents negative correlation, and X represents no statistically significant correlation (*P* < 0.001).(B) Alteration events for 29 m6A regulators and heatmap of clinicopathological information of samples with alteration of 29 m6A regulators in TCGA cohort.

Figure S6. m6A regulators significantly correlated with OS.

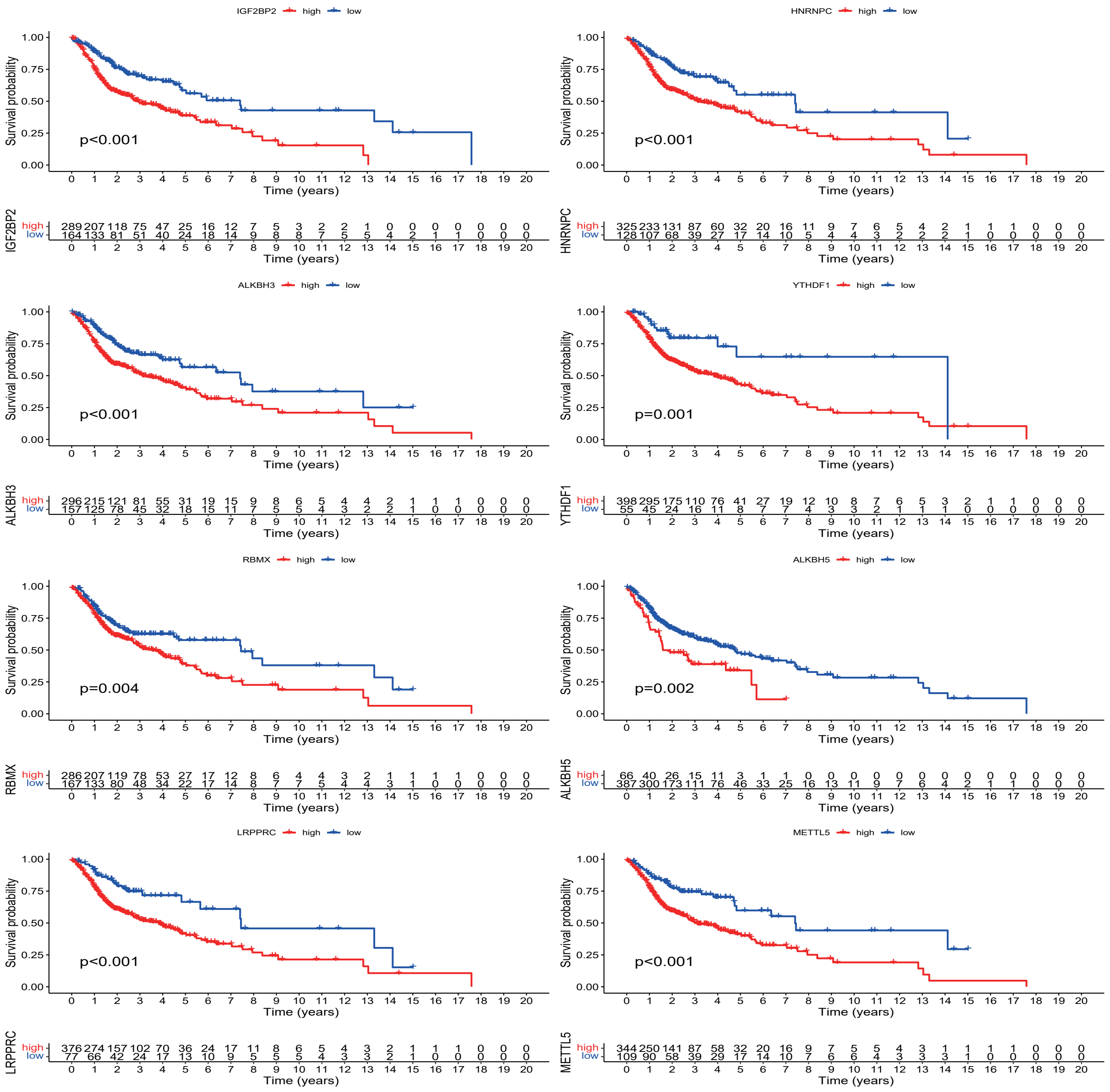

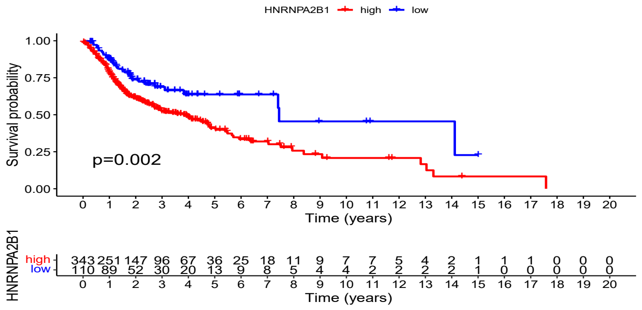

Kaplan-Meier curves of m6A regulators with statistical difference (P < 0.05) in the HNSCC patient cohort in TCGA.

Figure S7. GSEA for composite subgroups by GO, KEGG and Hallmark gene sets.

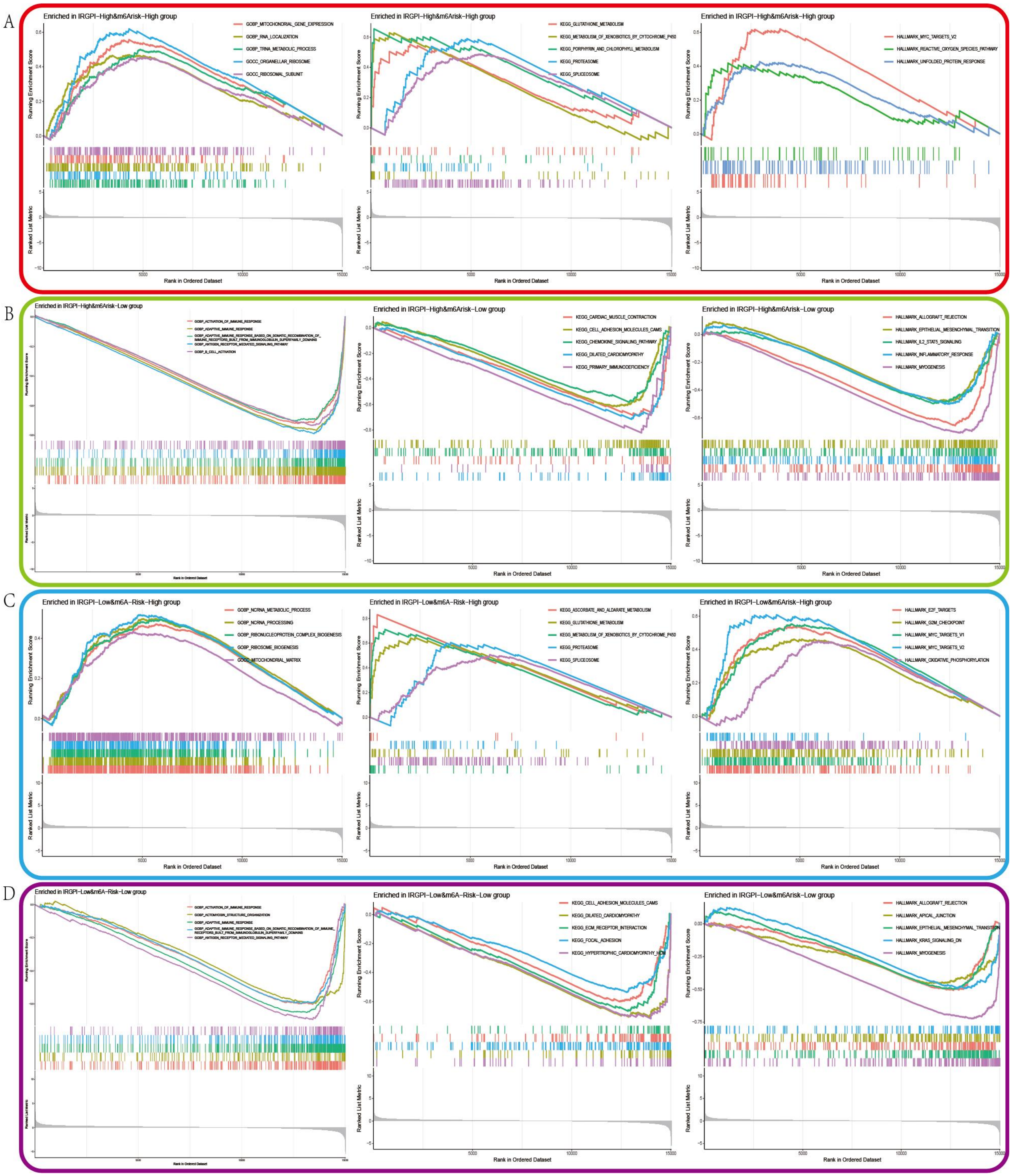

(A) GSEA for subgroup A by GO, KEGG and Hallmark gene sets ($P < 0.05$). (B) GSEA for subgroup B by GO, KEGG and Hallmark gene sets ($P < 0.05$). (C) GSEA for subgroup C by GO, KEGG and Hallmark gene sets ($P < 0.05$). (D) GSEA for subgroup D by GO, KEGG and Hallmark gene sets ($P < 0.05$).

Figure S8. Kaplan-meier curves of tumor infiltration of 22 immune cell types in TCGA.

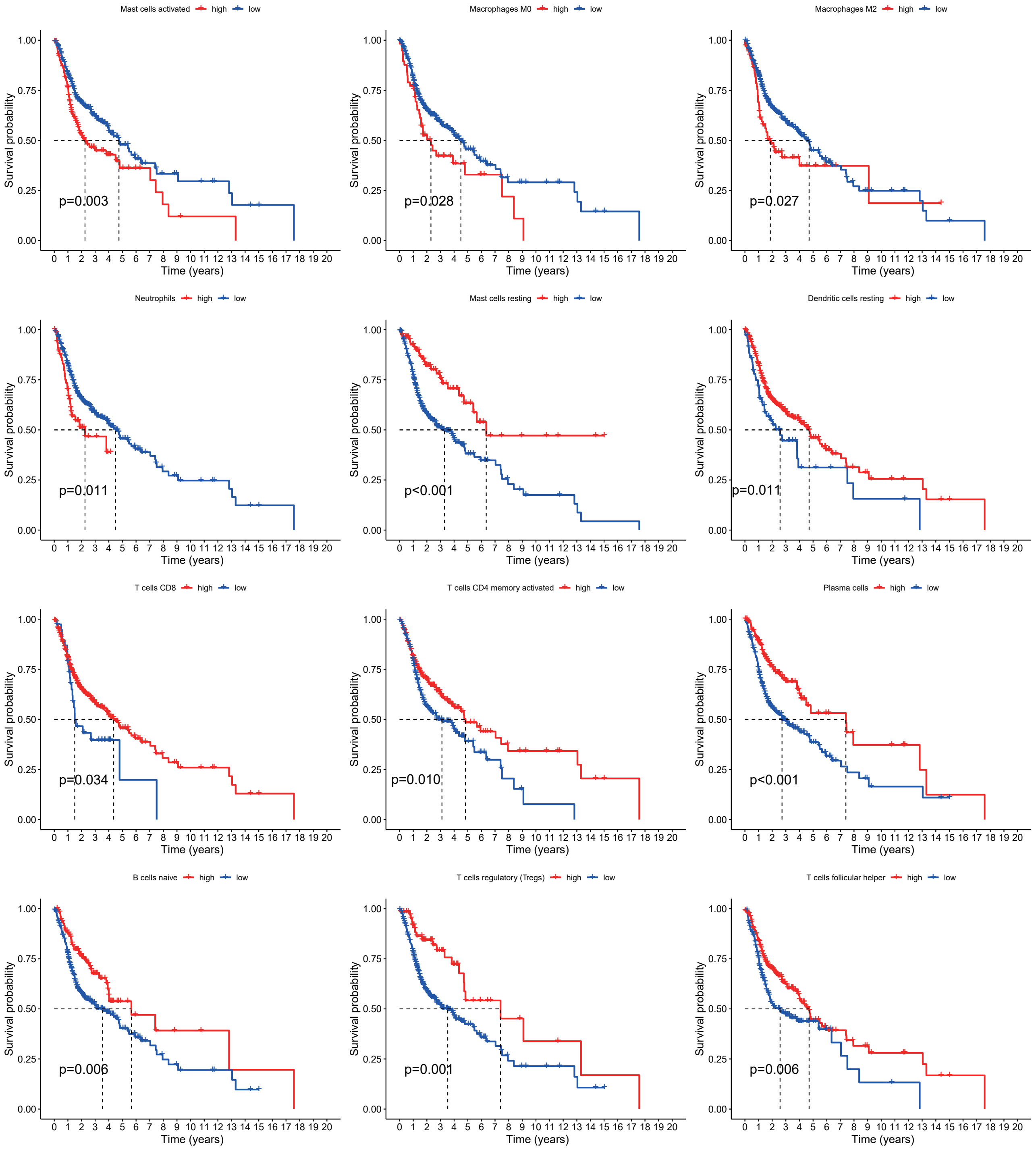

Kaplan-Meier curves of the immune cell infiltration with statistical difference ($P < 0.05$) in TCGA cohort.

Figure S9. Kaplan-meier curves of immune and molecular function signatures in the TCGA cohort.

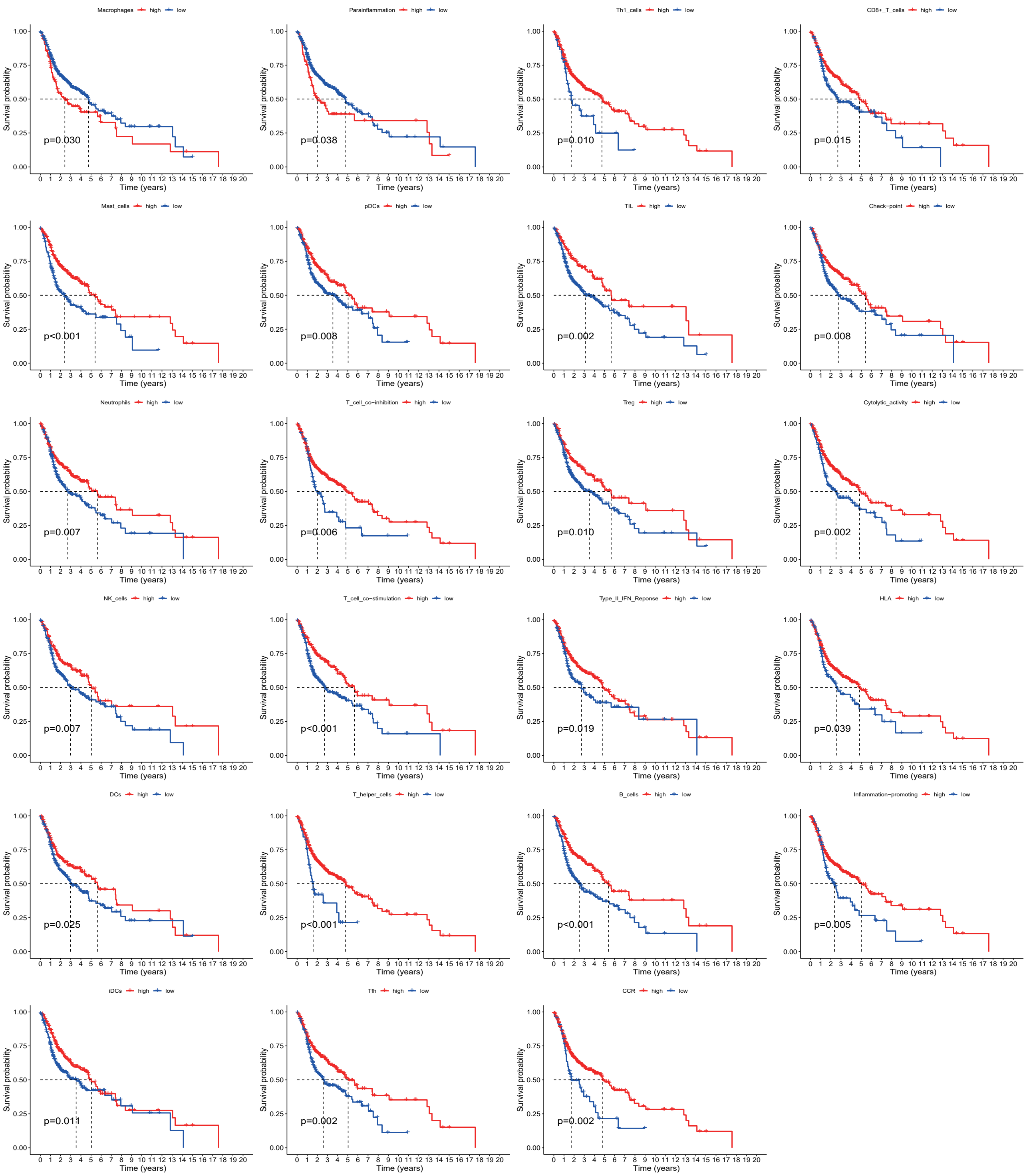

Kaplan-Meier curves of the immune and molecular function signatures with statistical difference (P < 0.05) in the HNSCC patient cohort in TCGA.

Figure S10. Scores derived from the TIDE database with significant differences in IRGPI and m6A risk score subgroups .

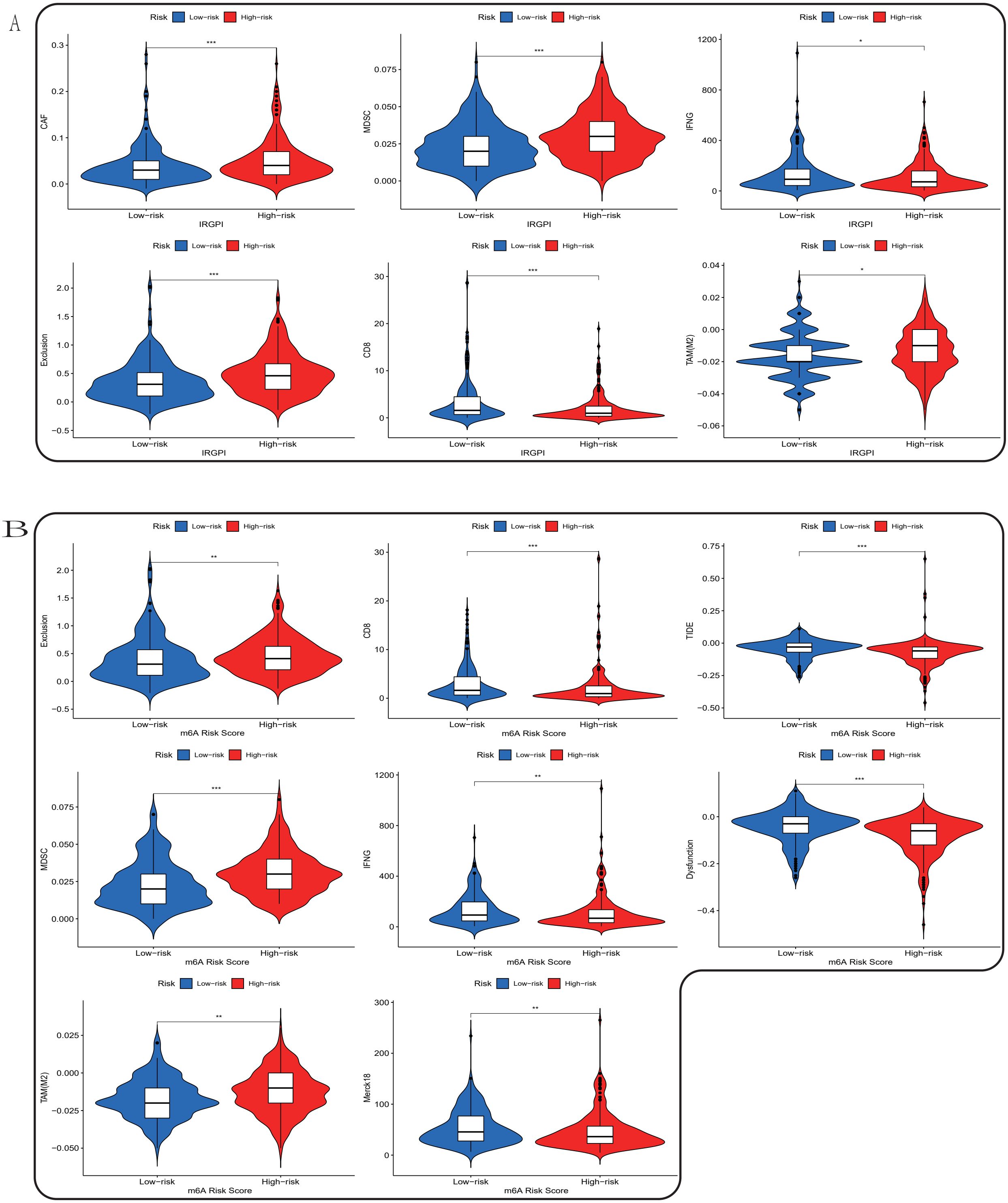

(A) IFNG, CD8, Exclusion, MDSC, CAF, and TAM (M2) scores in different IRGPI subgroups (Wilcoxon test, * P<0.05; ** P<0.01; *** P<0.001). (B) TIDE, IFNG, Merck18, CD8, Dysfunction, Exclusion, MDSC, and TAM (M2) scores in different m6A risk score subgroups (Wilcoxon test, * P<0.05; ** P<0.01; *** P<0.001).
